# The Fallacies of Crude Statistics

**Published:** 1895-10-12

**Authors:** 


### THE FALLACIES OF CRUDE STATISTICS.

If it were not that our faith in crude statistics is always tempered by the thought that some "correction" may have been omitted, we should have good ground for being appalled by the data in regard to the increase of cancer during the past fifty years which are detailed in the "Liverpool Medico-Chirurgical Journal" by Mr. Roger Williams, who is well known to have given considerable attention to this subject.

That in England and Wales over 18,000 more deaths per annum were caused by cancer in 1893 than in 1840 would of itself be a sufficiently startling statement were it not at once corrected by our knowledge of the increase of population during the interval between those dates. But Mr. Roger Williams puts the matter in a more convincing light when he gives the ratios of the cancer deaths to the population living and also to the deaths from all causes in each year. For every million persons living in 1840 we are told that 177 died of cancer, while in 1893 the number so dying per million was 711.

Again, the proportion of cancer deaths to total deaths, which in 1840 was only 1 to 129, had risen in 1893 to 1 cancer death to every 27 deaths from other causes. This increase is so enormous that one naturally asks on the one hand what possible correction can take away the gravity of the facts so disclosed; and, on the other, what possible value can be attached to crude death-rates from any cause if so enormous a discrepancy can be explained away. About two years ago a most careful and elaborate paper on this subject appeared in the "Proceedings of the Royal Society," by Mr. King and Dr. Newsholme, showing how largely the alleged increase of cancer must be discounted by the facts (1) that more people now live on into the cancer age than in years gone by; (2) that registration of the cause of death has been much more carefully carried out of late years; and (3) that accuracy of diagnosis has so far improved that the disease is better recognised—so much better, in fact, that coincidently with the apparent increase in the frequency of cancer there has been a great diminution in the number of deaths from ill-defined causes.

Many facts also are given which appear strongly to corroborate the conclusion at which these authors have arrived, viz., that cancer is not increasing. Nevertheless, it is clear that what they offer is explanation rather than disproof of the apparent increase, and while everyone will admit that there are difficulties in believing that cancer has increased so enormously as it is stated to have done by Mr. Roger Williams, there are difficulties quite as great in admitting that such positive data as figures taken from the Registrar-General's Returns can be so easily explained away.

We are, in fact, in this dilemma, that if we are to allow that the statistics of Mr. Roger Williams, showing increase of cancer, are not to be accepted, we must apply the same methods of correction to the statistics of every other disease that appears to be increasing, and entirely decline to argue upon the crude returns of the Registrar-General.

Truth is seldom all on one side, and the old adage as to the safety of a middle course probably applies to this question of the increase of cancer. But as regards crude statistics, and the many arguments founded on them, it must be admitted that these are often of but small value.

As an illustration of this, we may mention a point to which attention has recently been drawn by the medical officer of health for Brighton, viz., the necessity of considering the influence of a rising or falling birth-rate whenever the meaning of a death-rate is being considered. One of the most remarkable features of the vital statistics of recent years has been the lowering of the birth-rate, which, between 1883 and 1893, declined 9 per cent. The effect of this has been to diminish the proportion of young children in the population, and thus to lower the mortality, for the death-rate of children under five years of age is greater than at any subsequent period of life up to 65 years of age. It thus happens that while the crude statistics tell us that the death-rate is lessening year by year and so lead to anticipations of long life, this lessened death-rate in part arises from there now being a smaller infantile population, and so far as it is so produced has no relations at all to improved health conditions. The age and sex distribution also varies in every town, to such an extent in fact that proportionate corrections have to be applied before anything approaching a true general death-rate can be given; and if such corrections are necessary for the estimation of true death rates from statistics of mortality derived from all causes grouped together, they are infinitely more necessary in the consideration of the prevalence or fatality of individual diseases which affect some sections of the population more than others. There are few "fact heaps" so pregnant with untruth, while in themselves so absolutely true, as the crude numbers given in the Registrar-General's Returns, and it behoves people to exercise the greatest caution in accepting any general deductions from them.

